# The Role of Organizational Citizenship Behavior and Gender between Job Satisfaction and Task Performance

**DOI:** 10.3390/ijerph18189499

**Published:** 2021-09-09

**Authors:** Giulia Casu, Marco Giovanni Mariani, Rita Chiesa, Dina Guglielmi, Paola Gremigni

**Affiliations:** 1 Department of Psychology “Renzo Canestrari”, University of Bologna, 40127 Bologna, Italy; marcogiovanni.mariani@unibo.it (M.G.M.); rita.chiesa@unibo.it (R.C.); paola.gremigni2@unibo.it (P.G.); 2 Department of Education Studies “Giovanni Maria Bertin”, University of Bologna, 40127 Bologna, Italy; dina.guglielmi@unibo.it

**Keywords:** job satisfaction, organizational citizenship behavior, task performance, path analysis, mediation, moderation, gender

## Abstract

Job satisfaction (JS) is an indicator of individual psychosocial health. Consistent evidence showed that voluntary extra-role behavior in organizations, namely organizational citizenship behavior (OCB), can also contribute to individual psychological health. JS has been found to positively influence employees’ OCB, and both JS and OCB have been found to predict employees’ task performance (TP). The purpose of this study was to investigate whether employees’ OCB mediates the relationship of JS with TP, taking into consideration gender as a potential moderator, and other sociodemographic and work-related characteristics as confounding variables. A total of 518 employees, 54.6% women, aged 19–66 years with a mean age of about 36 years, completed measures of JS, OCB, and TP. Results showed a partial mediation of OCB in the JS-TP relationship, which was invariant across gender. A potential practical implication of findings is that human resource managers and practitioners might ultimately benefit male and female employees’ well-being as well as the organizations’ productivity by developing targeted individual- and group-level trainings and interventions to enhance JS and OCB.

## 1. Introduction

### 1.1. Job Satisfaction as a Dimension of Well-Being

In recent years, an emphasis has been placed on psychological well-being across multiple life domains, including the workplace [1]. The well-being of people in the workplace has important implications not only in terms of individual physical, mental, and social health but also in terms of its potential positive economic impact [2]. As a result, many attempts have been made to understand factors involved in employees’ well-being [1]. Within the framework of positive psychology, Parker and Hyett [3] identified job satisfaction (JS) as a main dimension of well-being in the workplace that may cover both hedonic (i.e., pleasure-focused) and eudemonic (i.e., meaning-focused) well-being principles [4]. Accordingly, they referred to JS as the people’s view of their work as a positive, rewarding experience that increases self-worth, gives direction and meaning to life, and fosters skills [3]. The importance of JS is linked to its positive role in improving individual and social well-being on the one hand [5] and increasing work productivity on the other hand [6,7].

### 1.2. The Link of Job Satisfaction with Task Performance

Productivity is a relevant outcome from an organization perspective. A crucial aspect of productivity is individual job performance, defined as a set of individual actions that are relevant to the goals of organizations [8]. Task performance (TP) is one of the most investigated dimensions of job performance [9] due to its contribution to the organization’s technical core [8]. It refers to the effectiveness with which workers carry out the tasks that are formally part of their job [8].

Cross-sectional studies reported a positive direct effect of JS on job performance in general [10,11]. A meta-analysis of longitudinal studies found a significant positive effect of JS on TP and no evidence for a reverse effect [12]. A more recent longitudinal study [13] confirmed that more satisfied workers have higher TP over time. In addition, it found support for a reciprocal model, where JS promotes TP, which, in turn, contributes to employees’ JS [13].

The positive effect of JS on employee performance has been attributed to the large influence of JS on the workers’ motivation, which has a strong impact on their productivity [14]. This relationship is consistent with the perspective of social psychology suggesting that attitudes such as JS can act as causes of behaviors such as job performance [15].

### 1.3. The Role of Organizational Citizenship Behavior

In the last decades, organizational citizenship behavior (OCB) has been a major construct in the field of organizational psychology for its positive impact on organizations and their employees [16,17]. OCB is a prosocial behavior defined as the employees’ voluntary performance of extra-role tasks that are not recognized by the organization’s formal reward system [18]. Examples of OCB include helping a newcomer become familiar with the new role and office, assisting a colleague with a specific task, or working overtime without expectation of getting paid [19].

OCB is recognized as a primary component of individual- and organizational-level effectiveness [20]. Studies have shown that OCB contributes to employees’ well-being by helping them work together [21,22] and has a positive impact on organization productivity by enhancing employees’ TP [23,24,25,26,27]. Therefore, TP could be seen as an outcome of employees’ OCB.

Equally important is to identify the antecedents of OCB. A large literature has shown that higher levels of JS may promote employees’ involvement in OCB [16,17] suggesting that JS could be seen as an antecedent of OCB. The social exchange theory proposes that the positive or negative behavior exhibited by employees is a response to the treatment they received from their employers [28]. A positive employer-employee relationship would elicit, in the employees, reactions such as trust and JS that, in turn, may stimulate them to engage in positive organizational behaviors like OCB [28].

Altogether, many studies have shown that employees’ TP can be positively influenced by both OCB [23,24,25,26,27] and JS [9,12,29], and other studies have shown that OCB can be promoted by JS [16,17]. Thus far, however, only a few studies have considered the three dimensions simultaneously within a mediation model. Indeed, two studies found that JS had significant positive effects on TP using OCB as an intervening variable [30,31] but results were not reported in terms of total or partial mediation.

### 1.4. The Role of Gender

An important characteristic that should be taken into consideration in addressing the relationships between JS, OCB, and TP is gender. At work, women, compared to men, are paid less, receive fewer promotions [32], and are less present in the EU labor market, with a gender employment gap of 11.7% in 2019 [33]. Despite being less paid and facing worse job conditions, women reported slightly higher OCB levels than men [34]. However, as far as JS and TP are concerned, the literature reports mixed findings: women showed higher JS and TP than men in some studies [34,35,36], while little or no gender differences were found in other studies [37].

A recent cross-national study in 32 European countries [38] argued that the gender-JS gap is attributable to experiences in early stages of life: lower job expectations as a result of early exposure to gender unequal socio-economic norms could explain women’s higher JS [38,39]. On the other hand, the absence of a gender gap in work outcomes found in some studies may imply that women work harder than men, often losing their leisure time [40] and drawing help from various sources (e.g., childcare facilities) to deal with housework and childcare without decreasing their JS and job performance [41].

In studies that investigated the associations of JS and/or OCB with TP, gender has been mainly considered as a control variable, and rarely explored as a moderator. In those few studies that addressed gender-specific associations, gender moderated the relationship of JS with TP rated by the supervisor [42] but had no moderating effect when an objective indicator of job performance was used [43]. Thompson et al. [44] found that the positive relationship between OCB and perceived organization support, which is positively related to JS [45], was stronger among men than women. Finally, an Iranian study reported gender differences in the positive relationship between OCB and TP [46].

### 1.5. The Relation with Other Characteristics

Other sociodemographic and work-related characteristics should be taken into consideration as they were found to be associated with JS, OCB, and TP. A positive relationship between age and JS has been frequently reported [47,48], and greater self- and other-rated OCB have been observed in older employees [49,50,51]. A weak age-performance relationship also exists, with peak TP occurring at about 30 years [49,52]. Evidence of educational effects on JS is still inconclusive: some authors found a positive relationship [53], whereas others reported that educational level was weakly negatively related to JS [54]. Instead, as educational level increases, TP and engagement in OCB also increase [51,55]. Meta-analyses found no significant differences in JS between full- and part-time workers [56], and no relationship between working hours and TP [57]. However, full-time employees showed higher OCB than their part-time counterparts [58]. Finally, as organizational tenure increases, employees become less satisfied with their job [48], whereas meta-analytic findings indicate a positive, although weak, relationship of organizational tenure with both OCB and TP [59].

### 1.6. Research Questions and Hypotheses

This study aims to fill an existing gap in the literature about the relationship among JS, OCB, and TP by jointly relating all three variables for the first time.

The first association we were interested in was that from JS to TP, for the positive role of both dimensions in the work life of individuals and organizations [5,6,7]. We were then interested in the role of OCB in the JS-TP relationship because OCB also has been found to benefit both employees and organizations [17].

Based on the literature, we hypothesized that:

**Hypothesis** **1.** *JS is positively related to TP*.

This hypothesis is based on the fact that JS refers to a cognitive and/or affective evaluation of one’s job [60]. As such, it is central to the implementation of behaviors relevant to organizational goals, such as TP [6].

**Hypothesis** **2.** *The relation of JS to TP is partially mediated by OCB*.

**Hypothesis** **2a.** *JS encourages employee involvement that can manifest in the form of OCB [16]; thus, JS may be positively related to OCB*.

**Hypothesis** **2b.** *In turn, OCB promotes higher in-role performance [23,24,25,26,27] that can result in better TP [61]; thus, OCB may be positively related to TP*.

An additional research question in this study is whether the relationships among JS, OCB, and TP are moderated by gender or are invariant across gender. Research on differences in these associations between men and women is still scarce and findings are inconclusive [42,43,44,46]. Thus, we did not formulate specific directional hypotheses about the role of gender in our mediation model.

Finally, we considered the potential confounding effect of age, educational level, work status, and organizational tenure, based on meta-analytic findings that underscore their associations with JS, OCB, and TP [47,49,55,56,57,59].

## 2. Materials and Methods

### 2.1. Design and Procedure

This study had a correlational design. Data were collected before January 2020 through an online survey and involved a convenience sample from the general population. Inclusion criteria were an age of 18 years or older, the ability to understand Italian, and a working status as employee either full-time or part-time. Therefore, retired, unemployed, homemakers, students (except for working-students), and self-employed people were excluded. The researchers, with the help of 20 students and trainees, recruited the sample through an e-mail invitation with characteristics such as personalization of the invitation and direct access to the online survey placed at the bottom of the invitation [62]. This convenience sampling strategy was used to collect data from a variety of work sectors. The online survey was preceded by a detailed illustration of the scope of the research, the inclusion criteria for participants (i.e., currently working and not being self-employed), and the guarantee of anonymity. Each participant in the study clicked an informed consent button before starting to fill in the survey. Nine hundred invitations were sent. The study was approved by the Bioethics Committee of the University of Bologna (protocol number 71562).

### 2.2. Participants

**Figure 1:**
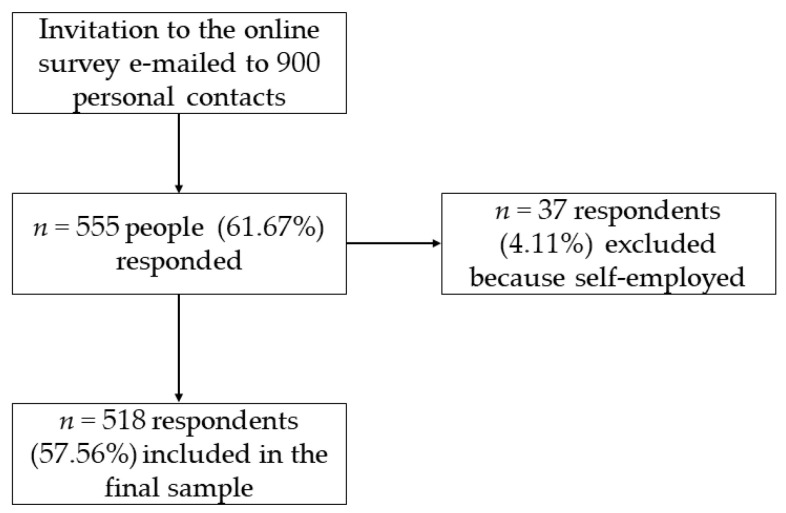
Flowchart of survey respondents.

Participants who completed the survey were 555 (61.67% response rate); however, 37 were excluded because they declared to be self-employed. Therefore, the final sample included 518 employees (Figure 1).

In the final sample, there was a slight prevalence of women and mean age was about 36 years (range 19–66 years). Educational level was categorized as low-to-medium (up to high school) or high (degree or post-degree). Most workers had a full-time job, and their organizational tenure had a median value of 4 years (range 1–520 months). Men and women did not differ in age, *F*(1, 516) = 1.36, *p* = 0.24, educational level, *χ^2^*(1) = 0.01, *p* = 0.94, or organizational tenure, *F*(1, 516) = 1.05, *p* = 0.31, but a larger proportion of women (33.57%) than men (14.47%) were employed part-time, *χ^2^*(1) = 25.05, *p* < 0.001. The participants’ characteristics are presented in Table 1.

The 37 excluded self-employed participants were 59.46% men, aged 25–66 years (*M* = 38.29, *SD* = 12.42). Educational level was 72.97% high, 86.49% had a full-time job, and their organizational tenure had a median value of 6 years (range 1–436 months).

### 2.3. Measures

The survey had a first section collecting sociodemographic information (i.e., gender, age, and level of education) and occupational characteristics such as employment status (employed, self-employed, retired, unemployed, homemaker, or students), work status (full-time or part-time), and permanence in the current organization (in months). The second part contained three self-report questionnaires measuring the psychological variables of interest. We included in the survey a limited number of subscales taken from multidimensional tools to avoid overburdening the respondents. For each variable, definitions and measures used in this study along with number of items are presented in Table 2. Content of the English version of selected subscales is provided in Appendix A.

JS was measured with the Work satisfaction subscale of the Workplace Well-being Questionnaire (WWQ) [2,3]. The WWQ measures aspects of employees’ well-being in the workplace using four subscales (Table 2). We selected the 10-item Work satisfaction subscale because the authors operationalized it as a dimension of well-being in the workplace (sample item: “Do your daily work activities give you a sense of direction and meaning?”). Items are rated on a 5-point scale from 0 = “not at all true” to 4 = “extremely true”. In the original WWQ, Work satisfaction subscale had all items loading on the same latent variable with standardized factor loadings between 0.49 and 0.85 [2] and showed good reliability, with a test-retest coefficient of *r* = 0.85 over a 4-week period [3]. The Italian version of the Work satisfaction subscale was obtained for the present study through translation and back-translation made by three independent bilingual researchers. Confirmatory factor analysis (CFA) used to test for structural validity showed good fit indices, *χ^2^*(35) = 71, *p* < 0.001; CFI = 0.99; RMSEA = 0.04; SRMR = 0.06, and standardized factor loadings between 0.48 and 0.90 (*p* < 0.001). Reliability in the present study was *ω* = 0.92 (95% CI 0.90–0.93).

OCB was assessed using the Italian version [63] of the Organizational Citizenship Behavior (OCB) scale [64]. The Italian OCB scale includes 15 items measuring three aspects of employees’ voluntary extra-role behavior, namely Altruism (e.g., “I help others who have heavy workload”), Civic virtue (e.g., “I attend meetings that are not mandatory, but important”), and Conscientiousness (e.g., “I respect company rules and policies even when no one is watching me”). Items are rated on a 7-point scale from 1 = “not at all true” to 7 = “completely true”. In the Italian validation study [63], the three OCB dimensions were strongly intercorrelated (*r*s of 0.53–0.58) and a global OCB score was computed, which showed a Cronbach’s *α* reliability coefficient of 0.84. A global score was used also in the present study, with a reliability of *ω* = 0.85 (95% CI 0.83–0.87).

TP was measured using the Task performance subscale of the Individual Work Performance Questionnaire (IWPQ) 1.0 version [65]. The IWPQ was developed based on a generic working population to be suitable across occupational sectors [66]. Its 18-item 1.0 version measures three dimensions of performance at work (Table 2). The 5-item TP subscale selected for the present study measures individuals’ performance on the tasks that are central to their job [8] (sample item: “I was able to perform my work well with minimal time and effort”). Items are rated on a 5-point scale from 0 = “seldom” to 4 = “always”, with a 3-month recall period. Reliability measured through the person separation index was 0.81 [65]. The Italian version of the TP subscale was obtained for the present study by a translation and back-translation made by three independent bilingual researchers. CFA used to test for structural validity showed acceptable fit indices, *χ^2^*(5) = 20.18, *p* = 0.001; CFI = 0.97; RMSEA = 0.07; SRMR = 0.03, and factor loadings between 0.42 and 0.72 (*p* < 0.001). Reliability in the present study was *ω* = 0.75 (95% CI 0.71–0.78).

**Table 2:** Definitions and measures of study variables.

Variable	Definition	Measure (N Items)	Subscales (N Items)
JS	Extent to which employees feel their work is fulfilling and increases their sense of self-worth [3]	Workplace Well-being Questionnaire (31) [2]	Work satisfaction (10), Respect for the employee (7), Employer care (7), Intrusion of work into private life (7)
OCB	A set of discretionary employee extra-role behaviors, which are not formally required in their job [18]	Organizational Citizenship Behavior scale (15) [63]	Altruism (6), Civic virtue (4), Conscientiousness (5) ^1^
TP	Proficiency with which employees carry out the core tasks of their job [8]	Individual Work Performance Questionnaire (18) [65]	Task performance (5), Contextual performance (8), Counterproductive work behavior (5)

JS, Job Satisfaction; OCB, Organizational Citizenship Behavior; TP, Task Performance. ^1^ An overall OCB score was computed by summing up the three subscale scores [63].

### 2.4. Data Analysis

Reliability of measures in this study sample was calculated with McDonald’s *ω* (> 0.70). CFA with maximum likelihood estimator was used to test for the structural validity of the Italian version of two questionnaires that were not previously validated in Italy. Harman’s single-factor test using CFA was performed to determine the potential threat of common method bias, which is present when a single latent factor accounts for more than 50 percent of the total variance of the measures used [67]. Gender effects on each of the study variables were tested using analysis of variance (ANOVA).

To test our hypotheses, we developed a partial mediation model where TP was predicted by JS both directly and indirectly, through the mediation of OCB. To preliminarily identify covariates to be included in the mediation model, associations of age, educational level, and job-related variables (i.e., work status and organizational tenure) with the psychological variables were examined using Pearson’s or point-biserial correlations, after recoding the dichotomous variable into 0–1.

Using path analysis with maximum likelihood estimation, we tested the direct and indirect associations of JS with TP. A bootstrapping procedure with 5000 samples and 95% confidence intervals (CIs) was applied to estimate and test the indirect association [68].

To explore the moderating role of gender, multi-group modeling was then applied. Wald test was used to examine whether the coefficients for each path in the model were equal among men and women, and the *χ^2^* difference test (Δ*χ^2^*) was used to examine whether indirect effects differed across gender [69].

We evaluated goodness of fit for both CFAs and path analysis using the following criteria: comparative fit index (CFI) ≥0.95, root means square error of approximation (RMSEA) ≤ 0.06, and standardized root mean square residual (SRMR) ≤ 0.08 [70]. CFAs and path analyses were performed using Mplus 7.2 (Muthén & Muthén, Los Angeles, CA, USA). All other analyses were conducted using IBM SPSS version 26 (IBM Corp., Armonk, NY, USA). Significance level was set at *p* < 0.05.

## 3. Results

Results of the single-factor test for potential threat of common method bias showed that a single-factor model accounted for only 20.39% of the total variance. Fit indices indicated poor fit: *χ^2^*(405) = 3655.92, *p* < 0.001; CFI = 0.52; RMSEA = 0.12; SRMR = 0.15. The *χ^2^* difference test also indicated that the three-factor model corresponding to the three measures used was superior to the single-factor model, Δ*χ^2^*(3) = 1993.20, *p* < 0.001. Consequently, common method bias was not a critical threat to the hypothesized relationships.

ANOVA results indicated a gender effect with women scoring slightly higher than men in JS and OCB (Table 3), while nonsignificant gender difference was found in TP scores.

Preliminary correlations between psychological variables were all positive and significant, *p* < 0.001. TP correlated *r* = 0.37 with OCB and *r* = 0.23 with JS, while OCB correlated *r* = 0.26 with JS. Among the potentially confounding variables (Table 4), age and organizational tenure were significantly associated with the mediator and outcome variables and also with each other, *r* = 0.73. Thus, they were included as covariates in the mediation model.

**Figure 2:**
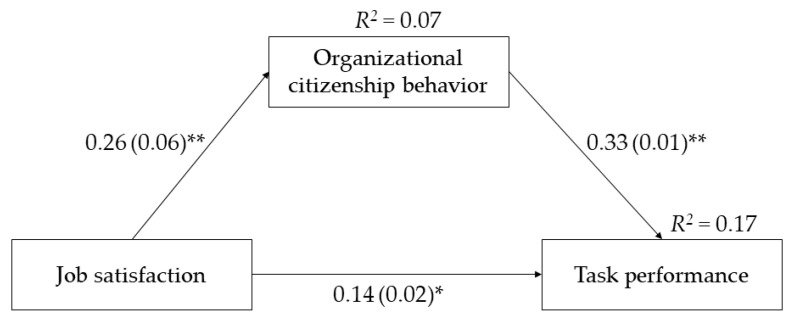
The mediation model. Standardized path estimates are displayed. Standard errors are in parentheses. Covariates are omitted from the figure for clarity. * *p* < 0.01; ** *p* < 0.001.

The hypothesized partial mediation model (Figure 2) yielded excellent fit, with *χ^2^*(2) = 2.83, *p* = 0.24; CFI = 0.99; RMSEA = 0.03; SRMR = 0.02. Higher JS was associated with higher OCB and thus with higher TP, with a significant indirect effect (estimate = 0.08, SE = 0.02, 95% CI 0.05–0.12). The direct positive association of JS with TP was also significant, indicating partial mediation. Among the covariates, only organizational tenure was significantly associated with TP (estimate = 0.13, SE = 0.01, *p* = 0.04).

Wald tests in multi-group analyses indicated that the mediation model was invariant across gender. Table 5 shows the standardized estimates of the different paths. Indirect effects estimates were 0.10 (SE = 0.03, 95% CI 0.05–0.15) for women, and 0.06 (SE = 0.02, 95% CI 0.01–0.10) for men. The difference was nonsignificant, Δ*χ^2^*(1) = 1.86, *p* = 0.17, indicating no moderated mediation.

## 4. Discussion

The present study aimed to augment knowledge of the relationships among three of the most important and popular constructs of psychology applied to the workplace: job satisfaction (JS), organizational citizenship behavior (OCB), and task performance (TP). A mediation model was tested in which the relationship between JS and TP was partially mediated by OCB. To the best of our knowledge, this was the first study to simultaneously relating JS, OCB, and TP using a mediation model. Noteworthy, we also tested for the potential moderator effect of gender in this mediation model and controlled for the effect of age and organizational tenure as they were significantly associated with OCB and TP.

Overall, the results provided support to our hypotheses. Consistent with Hypothesis 1, higher JS was directly associated with higher TP. As postulated in Hypothesis 2, this positive association was also indirect: higher JS was linked to higher OCB, which in turn was related to higher TP.

We cannot directly compare our results with the literature because mediation models linking JS, OCB, and TP had not been tested before. However, we can compare each path of our model with findings of previous studies.

The path between JS and TP is in line with Riketta’s meta-analysis of longitudinal studies [12], where the positive effect of JS on TP was weak (*β* = 0.06) but statistically significant as in the present study. As for the role of OCB, several correlational and longitudinal studies reported that OCBs are the informal modes of collaboration that employees adopt as a function of JS and perceived fairness [16,17,27]. Our significant and positive path between JS and OCB is in line with those findings. In their structural equation models, Wayne et al. [25] and Wang and colleagues [26] found a strong relationship (*β* = 0.80 and 0.77, respectively) between OCB and TP. This relationship was weaker in our study, but still considerable and statistically significant.

In summary, our findings are substantially in line with evidence that more satisfied workers show higher in-role (e.g., TP) and extra-role performance (e.g., OCB) [9,12,13,16,17,29,30,31]. The novelty of our study is that OCB partially mediates the effect of JS on TP. In other words, individual TP is not simply explained by individual self-perceptions of well-being in the workplace (JS) but there is also an extra contribution given by engagement of the individual in organizational prosocial behavior (OCB) that is stimulated by such a perceived well-being.

Our results appear more consistent if we consider that they were net of covariates such as age and organizational tenure, which were indicated as two primary time metrics in job satisfaction research [48]. Both variables had positive, weak bivariate correlations with OCB and TP, in line with previous findings [49,50,51,59]. However, in the mediation model, only the path coefficient from organizational tenure to TP was statistically significant. As for the other potential confounding variables considered, education and work status were not significantly associated with any of our study variables. This seems coherent with meta-analytic evidence of effect sizes close to zero when examining the correlation of educational attainment with JS [54] and with self-rated OCB and TP [55]. Additionally, it is in line with meta-analytic studies pointing to the lack of a relationship between working status and both JS and TP [56,57]. The lack of a significant association between work status and OCB in the present study could be attributed to our sample, as three quarters of the participants were employed full-time.

Regarding the effect of gender, we found that women were more satisfied with their job and engaged in more OCB than men, in line with previous evidence [34,35,38], while we found the same level of TP across genders as in a previous research [37] but different from other studies [34,35,36].

When testing the moderating effect of gender within our mediation model, multi-group analyses showed that all the relationships in the model were invariant across gender. Thus, in the present study, gender did not moderate the associations of JS and OCB with TP. Again, we cannot directly compare our results with previous studies; however, the gender-invariant association between JS and TP found in our study was in line with findings by Callaghan et al. [43], who adopted an objective measure of work productivity. Instead, our results differed from those by Nasir et al. [42,46], who found a moderating effect of gender in the relationship between JS and TP and between OCB and TP using supervisor ratings of TP. Future studies are therefore needed to explore the moderating role of gender in the JS-OCB-TP relationship using multisource job performance measurement.

### Limitations

The current study has several limitations that could be addressed in future research. First, the correlational design does not allow to determine cause and effect relationships. Therefore, although we found that common method bias was not a concern in our study, longitudinal studies are needed to confirm the effects of JS and OCB on TP and to investigate the potential bidirectional relationships between variables.

Second, to limit respondent burden, we measured JS and TP using only two subscales selected from the WWQ [2] and IWPQ [65], respectively. Some researchers warned against subscale extraction for self-report measures, as grouping items by their underlying factor might affect subscale means and intercorrelations [71]. However, more recent studies indicated that administering grouped scale items did not impact the psychometric functioning of psychological tools, suggesting that subscales can be extracted as necessary [72,73]. Based on this, we preliminarily checked the validity and reliability of JS and TP subscales in this study. For both JS and TP, CFAs supported a one-factor model with adequate model fit indices and reliability coefficients, in line with validation studies of the original multidimensional tools [2,3,65,66].

Third, we measured OCB and TP on the basis of self-reports. Although meta-analytic evidence supports the use and validity of self-rated OCB [74], using both self- and other-ratings has been recommended for TP [74,75]. Therefore, research adopting mixed-rating sources for a better measurement of OCB and TP is needed to replicate our findings and rule out any potential issues. Additionally, we only used quantitative data. It is thus advisable that future studies augment the analyses and findings of this study by adopting qualitative and mixed-method approaches, which would facilitate more in-depth knowledge of the relationships among JS, OCB, and TP.

Fourth, the generalizability of this study’s findings was limited by the convenience nature of our sampling strategy as well as by the use of the Internet to recruit the sample and administer the survey [76].

Finally, further variables that we have not considered might have an impact on job performance and a role in our mediation model. Future research could test the effect of work characteristics (e.g., occupation type, income levels, job security, leadership style) and organization-based attitudes (e.g., trust in the organization, organizational identification) on job performance, and include possible moderators like job content (in the perspective of TP), and organizational culture (in the perspective of OCB) [77,78,79,80].

## 5. Conclusions

The present study added a contribution to the stream of research that investigates the role that employees’ well-being at work, expressed through job satisfaction, may have on specific work-related outcomes such as involvement in organizational life and task performance.

Based on our research findings, different managerial implications can be considered. First of all, for managers, it becomes useful to monitor a wide range of employees’ behaviors not limited to those most closely related to specific role needs of the organization’s members. Managers and organizations interested in improving job performance are encouraged to monitor employees’ job satisfaction and organizational citizenship behavior due to their potential predictive effect on task performance. By collecting self-report data within the organization, it is possible to focus on specific aspects of job satisfaction that can be improved. The information collected can help managers provide employees with training and interventions to promote job satisfaction and prosocial behaviors that, in turn, may enhance organizational outcomes [81]. These activities can be carried out at both individual and group levels. At the individual level, human resource managers can operate on the one hand by enhancing incentive systems and benefits, and on the other hand, by developing training strategies based on individual strengths, such as self-efficacy, resilience, and stress management skills [82,83]. At the group level, previous studies have shown that job satisfaction can be enhanced through more effective relationships among team members [84]. Collaboration and cooperation among co-workers have been seen as crucial factors in predicting employees’ prosocial behaviors that contribute to organizational effectiveness [83]. Thus, it may be helpful to implement team-level trainings addressing relevant group aspects, such as communication, social interactions, and cooperation with the aim of improving prosocial behaviors.

For years, scholars have tried to highlight the components of individual performance at work [8,85], paying, however, little attention to how these are connected to each other and can create positive synergies. Future research may concentrate on better understanding the mechanisms behind organization and employee outcomes by testing more complex explanatory models. Further evidence should be collected to explain the relationships among individual psychological and behavioral variables in the workplace. This would help human resource managers and practitioners target their intervention strategies, which may ultimately provide benefits to male and female employees’ well-being as well as to the organizations’ productivity.

## Figures and Tables

**Table 1:** Participants’ characteristics (*N* = 518).

Characteristic	
Age, mean (*SD*)	36.30 (12.35)
Gender, *n* (%)	
Men	235 (45.37)
Women	283 (54.63)
Education, *n* (%)	
Low-to-medium	279 (53.86)
High	239 (46.14)
Work status, *n* (%)	
Full-time	389 (75.10)
Part-time	129 (24.90)
Organizational tenure (months), mean (*SD*)	101.19 (118.45)

**Table 3:** Gender differences in psychological variables.

	Gender		ANOVA		
	Men (*n* = 235)	Women (*n* = 283)	*F* (1,516)	*p*	Cohen’s *d*
JS, mean (*SD*)	33.91 (8.45)	35.94 (8.45)	7.35	<0.001	0.24
OCB, mean (*SD*)	84.45 (11.75)	89.22 (10.33)	24.21	<0.001	0.43
TP, mean (*SD*)	19.66 (3.17)	19.26 (3.00)	2.16	0.14	0.13

JS, Job Satisfaction; OCB, Organizational Citizenship Behavior; TP, Task Performance.

**Table 4:** Correlations of JS, OCB, and TP with covariates.

	JS	OCB	TP
Age	0.07	0.12 *	0.14 *
Education	0.01	−0.02	−0.08
Work status	0.05	0.01	0.03
Organizational tenure	0.05	0.11 *	0.18 **

JS, Job Satisfaction; OCB, Organizational Citizenship Behavior; TP, Task Performance. * *p* < 0.01; ** *p* < 0.001.

**Table 5:** Multi-group analyses on the moderating role of gender.

Effect	Males	Females	Wald Statistic	*p*
Job satisfaction → Task performance	0.14 (0.06)	0.14 (0.06)	0.003	0.96
Job satisfaction → Organizational citizenship behavior	0.21 (0.06)	0.27 (0.06)	0.12	0.73
Organizational citizenship behavior → Task performance	0.29 (0.06)	0.36 (0.05)	3.22	0.07

Standardized estimates (standard errors) are reported.

## Data Availability

The data presented in this study protocol are available from the corresponding author on reasonable request.

## Appendix A. Items of the Survey Questionnaires

*Workplace Well-being Questionnaire (WWQ)—Work satisfaction subscale* [2]. *Answer from “Not at all true” (0) to “Extremely true”* (4).

Is your work fulfilling?

Do your daily work activities give you a sense of direction and meaning?

Does your work bring a sense of satisfaction?

Does your work increase your sense of self-worth?

Does your job allow you to recraft your job to suit your strengths?

Does your work make you feel that, as a person, you are flourishing?

Do you feel capable and effective in your work on a day-to-day basis?

Does your work offer challenges to advance your skills?

Do you feel you have some level of independence at work?

Do you feel personally connected to your organization’s values?

*Organizational Citizenship Behavior (OCB) scale* [63]. *Answer from “Not at all true” (1) to “Completely” (7)*.

I help others who have a heavy workload.

I do my job without constant requests from my boss.

I believe in giving an honest day’s work for an honest day’s pay.

I keep abreast of changes in the organization.

I attend meetings that are not mandatory, but important.

I am always ready to give a helping hand to those around me.

I attend functions that are not required but help the company image.

I read and keep up with organization announcements, memos, and so on.

I help others who have been absent.

I willingly help others who have work-related problems.

I take steps to try to avoid problems with other workers.

I do not take extra breaks.

I respect company rules and policies even when no one is watching me.

I guide new people even though it is not required.

I am one of the most conscientious employees.

*Individual Work Performance Questionnaire (IWPQ)—Task performance subscale* [65]. *Answer from “Seldom” (0) to “Always” (4), with reference to the past three months*.

I managed to plan my work so that it was done on time.

My planning was optimal.

I kept in mind the results that I had to achieve in my work.

I was able to separate main issues from side issues at work.

I was able to perform my work well with minimal time.
